# Stigmasterol prevents glucolipotoxicity induced defects in glucose-stimulated insulin secretion

**DOI:** 10.1038/s41598-017-10209-0

**Published:** 2017-08-25

**Authors:** Meliza G. Ward, Ge Li, Valéria C. Barbosa-Lorenzi, Mingming Hao

**Affiliations:** Department of Biochemistry, Weill Cornell Medical College, New York, NY 10065 USA

## Abstract

Type 2 diabetes results from defects in both insulin sensitivity and insulin secretion. Elevated cholesterol content within pancreatic β-cells has been shown to reduce β-cell function and increase β-cell apoptosis. Hyperglycemia and dyslipidemia contribute to glucolipotoxicity that leads to type 2 diabetes. Here we examined the capacity of glucolipotoxicity to induce free cholesterol accumulation in human pancreatic islets and the INS-1 insulinoma cell line. Glucolipotoxicity treatment increased free cholesterol in β-cells, which was accompanied by increased reactive oxygen species (ROS) production and decreased insulin secretion. Addition of AAPH, a free radical generator, was able to increase filipin staining indicating a link between ROS production and increased cholesterol in β-cells. We also showed the ability of stigmasterol, a common food-derived phytosterol with anti-atherosclerotic potential, to prevent the increase in both free cholesterol and ROS levels induced by glucolipotoxicity in INS-1 cells. Stigmasterol addition also inhibited early apoptosis, increased total insulin, promoted actin reorganization, and improved insulin secretion in cells exposed to glucolipotoxicity. Overall, these data indicate cholesterol accumulation as an underlying mechanism for glucolipotoxicity-induced defects in insulin secretion and stigmasterol treatment as a potential strategy to protect β-cell function during diabetes progression.

## Introduction

As of 2014, an estimated 422 million people have diabetes worldwide, with type 2 diabetes representing approximately 90% of the adult cases^1^. The number of individuals with diabetes and the chronic nature of the disease pose a serious health and economic challenge. Due to the enormity of this issue, there is an urgent need for strategies to reduce the development of the disease. Given that lifestyle changes can be just as effective as drug interventions^2^, exploring dietary modifications that may prevent onset of type 2 diabetes is a worthwhile endeavor.

Type 2 diabetes is marked by insulin resistance and pancreatic β-cell dysfunction. As peripheral tissues become insulin resistant, insulin producing β-cells residing in pancreatic islets can compensate by increasing mass and secreting more insulin. Once β-cells fail to compensate for insulin resistance, hyperglycemia and type 2 diabetes ultimately occurs. Several genetic and environmental factors predispose individuals towards type 2 diabetes^3, 4^. In addition, impaired metabolic states, such as metabolic syndrome and obesity, increase risk for type 2 diabetes^5^. However, the exact causes leading to type 2 diabetes are still under intensive investigation.

Elevated glucose and non-esterified (free) fatty acids, termed glucolipotoxicity, is a major contributor to the progression of type 2 diabetes after predisposing factors are established^6^. Glucolipotoxicity is shown *in vitro* to increase β-cell death and decrease insulin secretion^7–9^. The effect of glucolipotoxicity may be in part due to changes in lipid partitioning and reactive oxygen species (ROS) production^10^.

Cholesterol accumulation impairs β-cell function, while removal of excess cholesterol promotes β-cell health^11, 12^. Glucolipotoxicity may lead to increased cholesterol in β-cells as evidenced by studies performed in mouse and cell lines^10, 13, 14^. Whether intracellular cholesterol plays a role in glucolipotoxicity is unclear. It has been reported that palmitate alone increases total cholesterol in β-cells, but does not coincide with apoptosis^10^. Others have reported that apoptosis induced by free fatty acids was due to decreased cholesterol within the ER^15^.

Due to the link between excess cholesterol and β-cell dysfunction, therapies that reduce β-cell cholesterol may be anti-diabetic. Although statins, which are inhibitors of 3-hydroxy-3-methyl-glutaryl-coenzyme A reductase (HMG-CoA reductase, the rate-limiting enzyme for cholesterol synthesis), are best at reducing serum cholesterol in order to prevent cardiovascular diseases, they present a modestly increased risk for the onset of new diabetes cases^16–19^. Possible mechanisms include side effects that are independent of statin’s cholesterol-lowering property, such as a statin-specific immune response leading to insulin resistance^20^. Therefore, alternative ways to reduce cholesterol in β-cells are needed.

Phytosterols are found in plant foods and are analogous to cholesterol in mammals. Phytosterols have been of interest due to their well-documented ability to decrease serum LDL-cholesterol levels in humans^21, 22^, with the greatest reductions at 2.5 grams of phytosterols per day^23^. Given that the typical Western diet provides 200–400 milligrams of phytosterols per day^24^, we may not be consuming phytosterols at a level needed for maximum health benefits. Absorption of phytosterols is regulated by small intestinal ATP-binding cassette transporters that export phytosterols into the lumen^25^. Although the amount of phytosterols absorbed into serum is low, it can accumulate in tissues. A study by Alhazzaa^26^ showed that feeding rats phytosterols increases accumulation in all the internal organs measured except the brain.

Commonly consumed dietary phytosterols include stigmasterol, campesterol and sitosterol. Stigmasterol has been widely investigated for its anti-atherosclerotic cholesterol-lowering effects. Stigmasterol increases cholesterol efflux and decreases LDL-induced proinflammatory cytokine secretion, whereas campesterol and sitosterol have no beneficial effects^27^. The purpose of this study is to assess the capacity for stigmasterol, a common food-derived phytosterol with anti-atherosclerotic potential, to prevent β-cell dysfunction induced by glucolipotoxicity.

## Results

### Glucolipotoxicity reduces insulin secretion by inducing cholesterol accumulation

**Figure 1:**
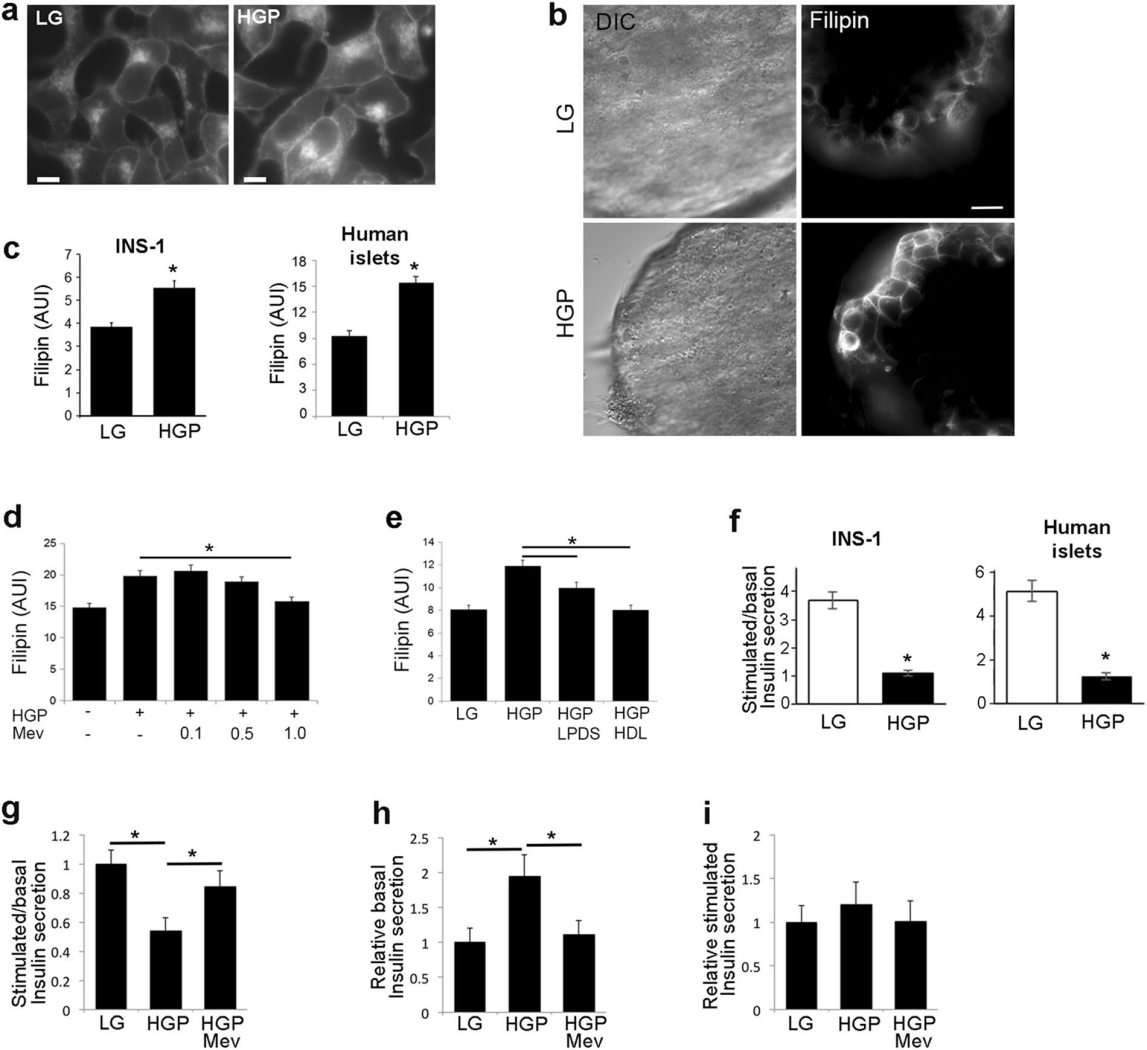
Glucolipotoxicity increases free cholesterol and decreases insulin secretion. ( **a**–**c**) HGP increases free cholesterol. INS-1 cells (**a**) or human islets (**b**) were treated for 72 h with control growth medium (LG), or HGP. Filipin per cell, indicating free cholesterol, was quantified from 5 independent experiments (**c**). DIC, differential interference contrast. AUI, arbitrary unit of intensity. Scale bars, 10 μm. (**d**) Mevinolin (Mev), an inhibitor of cholesterol synthesis, blocks HGP-induced increase in filipin staining in a dose-dependent manner. Cells were treated with HGP and Mev (μM) in LPDS growth medium for 72 h. (**e**) LPDS or HDL treatment blocks HGP-induced increase in filipin staining. Cells were treated with LG, HGP, HGP in LPDS medium (HGP + LPDS) or HGP and HDL in LPDS medium (HGP + HDL) for 72 h. (**f**) HGP decreases GSIS. Static GSIS assays of INS-1 and human islets were performed in cells treated for 72 h with HGP. (**g**–**i**) Mev treatment normalizes GSIS (**g**) by inhibiting HGP-induced basal insulin secretion (**h**) without affecting stimulated insulin secretion (**i**). All values are expressed as fold change relative to LG. n = 5 experiments. *p < 0.05.

Cholesterol excess is a possible contributing factor to β-cell failure. To determine if glucolipotoxicity treatment can increase free cholesterol within β-cells, INS-1 insulinoma cells and human islets were treated with HGP (30 mM glucose, 0.5 mM palmitate) or normal growth medium (LG) for 72 h. Filipin is a widely used fluorescent probe to measure free cholesterol in cells^28^. HGP treatment increased filipin staining in INS-1 cells and human islets (Fig. 1a–c). The result from INS-1 cells was consistent with other reports^10^. To our knowledge, this was the first time the effect of glucolipotoxicity on cholesterol in human islet was examined (Fig. 1b,c). Cholesterol accumulation may occur through increased uptake of LDL, increased biosynthesis, or decreased efflux to acceptors such as HDL. Treatment with mevinolin (Mev), which inhibits the rate-limiting enzyme of cholesterol biosynthesis HMG-CoA reductase, dose-dependently reduced cholesterol accumulation in cells exposed to HGP (Fig. 1d). Similarly, removing LDL as a source for cholesterol uptake by using lipoprotein-deficient serum (LPDS) in place of FBS or adding HDL to LPDS growth medium also inhibited HGP-induced cholesterol accumulation in INS-1 cells (Fig. 1e).

The increase in cellular free cholesterol was accompanied by reduced glucose-stimulated insulin secretion (GSIS) in both INS-1 cells and human islets treated with HGP (Fig. 1f). Consistent with an inhibitory role of elevated cholesterol in GSIS, GSIS was normalized when HGP-induced cholesterol accumulation was blocked by mevinolin (Fig. 1g). The effect of mevinolin treatment was accomplished through normalization of basal insulin secretion (Fig. 1h), with little effect on stimulated insulin secretion (Fig. 1i).

## Oxidative stress increases β-cell cholesterol

**Figure 2:**
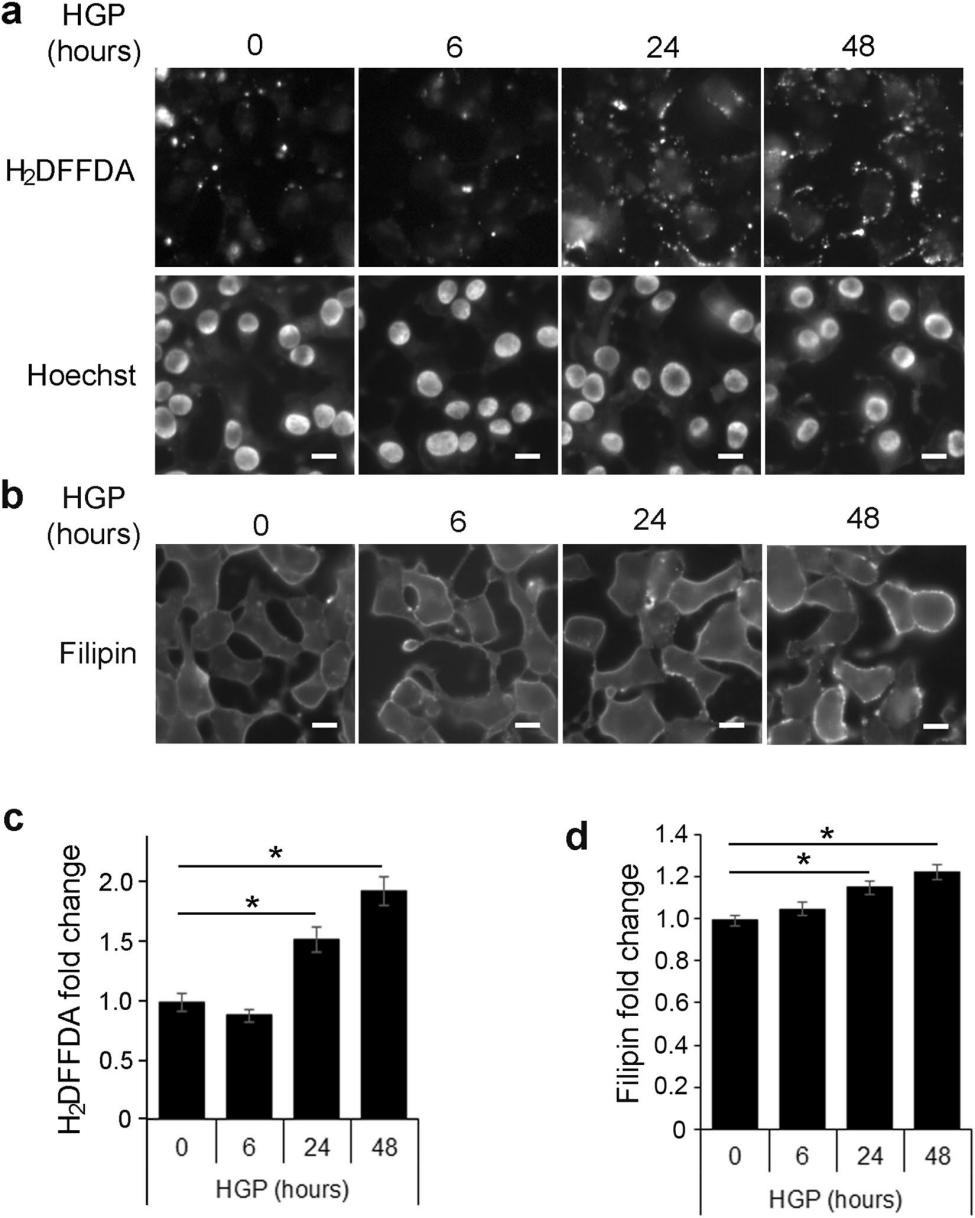
Glucolipotoxicity increases ROS in β-cells. (**a**,**b**) Timecourse for HGP-induced ROS production (**a**) and cholesterol accumulation (**b**). INS-1 cells were incubated for up to 48 h with HGP and imaged using carboxy-H_2_DFFDA (**a**) and filipin (**b**), along with Hoechst stain for cell counting. Scale bars, 10 μm. (**c,d**) Background-corrected fluorescence was measured for carboxy-H_2_DFFDA (**c**) and filipin (**d**) fluorescence. *p < 0.05.

Pancreatic β-cells are particularly susceptible to oxidative stress because they express low levels of intracellular antioxidants^29^. ROS has been linked to cholesterol accumulation in neurons, vascular smooth muscle cells, and hepatocytes^30–32^. To determine if ROS may be the underlying cause for HGP-induced cholesterol accumulation, INS-1 cells were incubated with HGP for time periods up to 48 h. As shown in Fig. 2a, HGP increased ROS production as estimated by carboxy-H_2_DFFDA staining. Carboxy-H_2_DFFDA becomes fluorescent when oxidized and is used as a general marker of ROS^33^. The increase in ROS corresponded to a rise in filipin staining (Fig. 2b). The most significant increase in ROS (Fig. 2c) and free cholesterol (Fig. 2d) both took place between 6 and 24 h after HGP treatment, suggesting that the rise in ROS and cholesterol may occur with similar time course.

**Figure 3:**
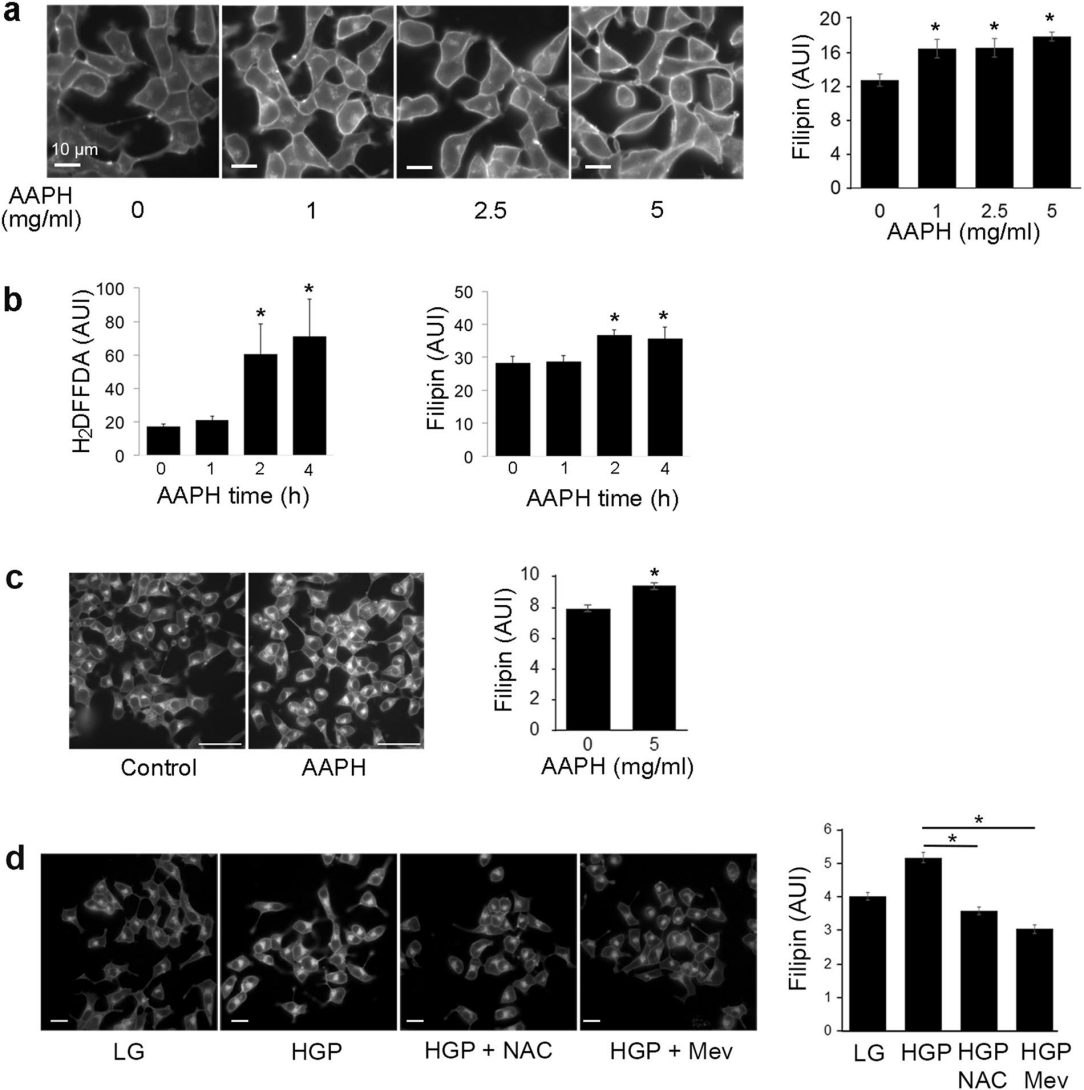
ROS induces cholesterol accumulation. (**a**) INS-1 cells were treated with AAPH in concentrations from 0 to 5 mg/ml for 4 h in serum-free media. Free cholesterol was measured using filipin. *p < 0.05 against control (no AAPH). Scale bars, 10 μm. (**b**) INS-1 cells were treated with 2.5 mg/ml AAPH for the different periods of time. Cellular staining of carboxy-H_2_DFFDA for ROS content and filipin for free cholesterol content was quantified. *p < 0.05 against control (no treatment). (**c**) Filipin staining of INS-1 cells incubated for 1 h with 5 mg/ml AAPH in serum free media. Scale bars, 50 μm. Filipin intensity per cell was quantified. *p < 0.05. (**d**) INS-1 cells were treated with LG, HGP, HGP + NAC (0.5 mM), or HGP + Mev for 48 h. Cells were imaged with filipin to measure free cholesterol. Scale bars, 20 μm. *p < 0.05 against HGP.

To determine if oxidative stress alone increases cholesterol accumulation in β-cells, INS-1 cells were incubated with AAPH, a water-soluble free radical generator used to produce ROS^34, 35^ (Supplementary Fig. 1). Cholesterol increased in INS-1 cells treated with varying concentrations of AAPH for 4 h in serum-free media (Fig. 3a). AAPH increased oxidation (H_2_DFFDA) and filipin staining with a similar temporal pattern, with the most significant rise occurring between one and two hours after AAPH addition (Fig. 3b). At the higher concentration range (5 mg/ml), AAPH was sufficient to increase free cholesterol labeled by filipin staining within 1 h (Fig. 3c). Esterified cholesterol labeled by lipidtox was also increased by AAPH during the same time frame (Supplementary Fig. 2). These data indicate that cholesterol accumulation in β-cells is sensitive to free radical generation. To further determine if ROS is the underlying cause for HGP-induced cholesterol increase in β-cells (Fig. 2), we treated INS-1 cells exposed to HGP with the antioxidant N-acetylcysteine (NAC) to prevent HGP-induced ROS production. Mevinolin (Mev) was included as a positive control of cholesterol depletion. As shown in Fig. 3d, NAC prevented an increase in filipin staining in HGP-treated INS-1 cells. Together with the results from AAPH, these data suggest that ROS stress can raise free cholesterol within β-cells, and the presence of antioxidants can prevent HGP-induced cholesterol increase.

### Stigmasterol reduces free but not esterified cholesterol

**Figure 4:**
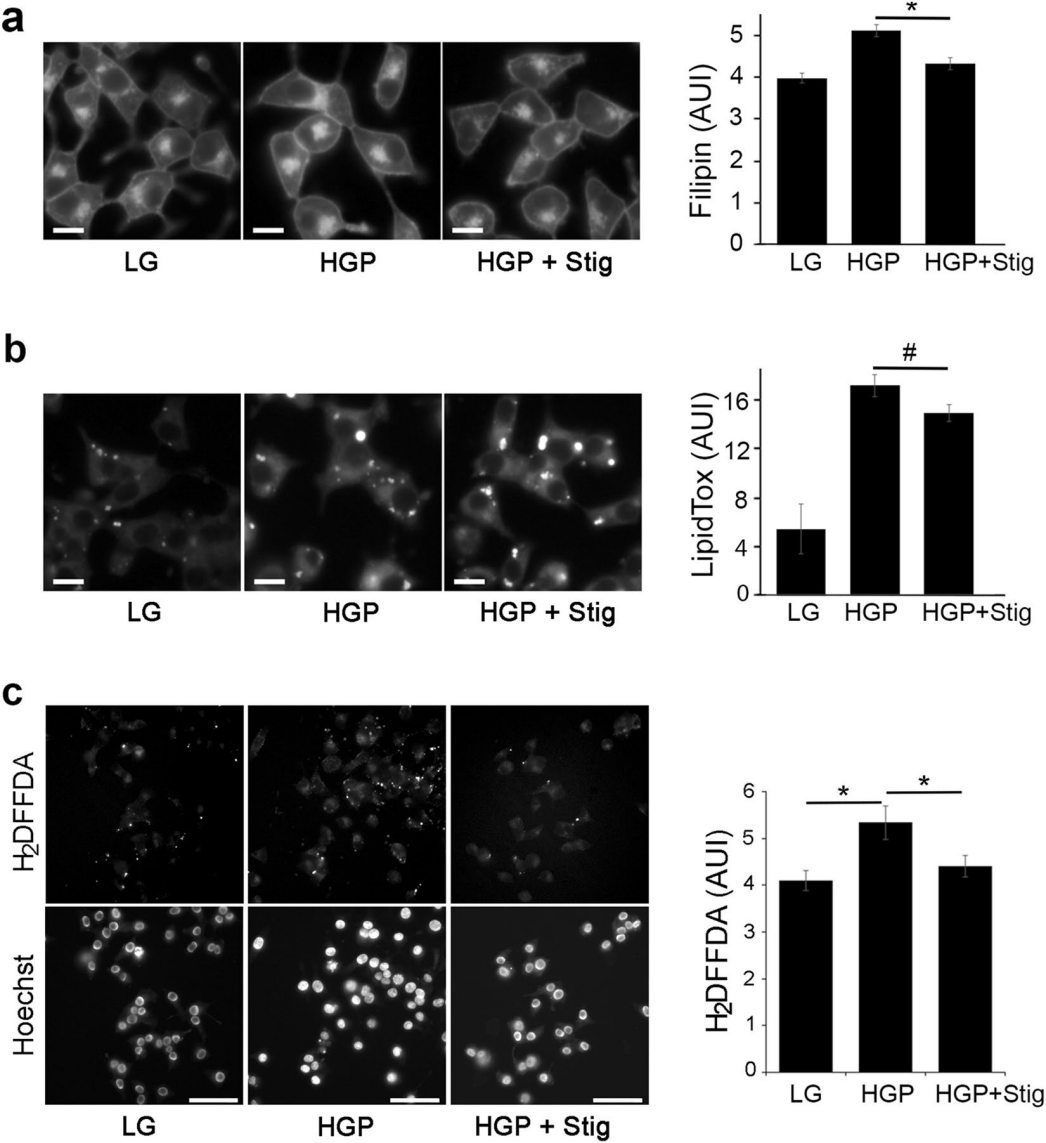
Stigmasterol treatment inhibits HGP-induced increase in free cholesterol and ROS. (**a**,**b**) Free cholesterol but not esterified cholesterol was reduced by stigmasterol. INS-1 cells treated for 72 h with LG, HGP, or HGP + stigmasterol (Stig) were stained with filipin (**a**) and lipidtox (**b**). Scale bars, 10 μm. *p < 0.05 and ^#^not significant against HGP. (**c**) ROS is reduced by stigmasterol. INS-1 cells were incubated in LG, HGP, or HGP + stig, and carboxy-H_2_DFFDA was used to measure ROS production. Carboxy-H_2_DFFDA fluorescence was quantified by normalizing to cell number (Hoechst staining). Scale bars, 50 μm. *p < 0.05.

Because glucolipotoxicity increases β-cell cholesterol, which is at least partially responsible for reduced GSIS observed in HGP-treated cells, we next tested whether including stigmasterol with HGP could rescue β-cell function. While there are studies of stigmasterol’s effects on lowering plasma cholesterol levels, how stigmasterol impacts cholesterol pools in cells is not well investigated. Cholesterol is found in either esterified or non-esterified (free) pools within the cell. We utilized filipin to measure free cholesterol and lipidtox to measure neutral lipids in INS-1 cells. As shown in Fig. 4a and b, both filipin and lipidtox staining increased with HGP treatment (HGP vs LG), indicating increased free cholesterol accumulation and conversion to cholesteryl esters. Stigmasterol decreased filipin staining due to HGP treatment (Fig. 4a, HGP + Stig vs HGP). The effect appeared to be specific to free cholesterol, as lipidtox staining, a measure of esterified cholesterol, did not change (Fig. 4b). LPDS treatment, on the hand, reduced both forms of cholesterol in INS-1 cells (Supplementary Fig. 3). At a high concentration, stigmasterol was as effective as LPDS at reducing free cholesterol, but remained ineffective at reducing esterified cholesterol (Supplementary Fig. 3).

### Stigmasterol decreases glucolipotoxicity-induced ROS production

Stigmasterol is reported to increase glutathione content in mice, indicating an antioxidative property for stigmasterol^36^. Because β-cells naturally have low levels of glutathione^29^, stigmasterol treatment could act by reducing the ROS that accompanies glucolipotoxicity. Increased carboxy-H_2_DFFDA fluorescence, an indication of elevated ROS, was found in INS-1 cells treated with HGP (Figs 2 and 4c, HGP vs LG). Interestingly, the addition of stigmasterol with HGP decreased the amount of carboxy-H_2_DFFDA fluorescence, consistent with the idea that stigmasterol could decrease ROS production in β-cells exposed to glucolipotoxicity (Fig. 4c).

### Stigmasterol alters expression of cholesterol homeostasis genes

**Figure 5:**
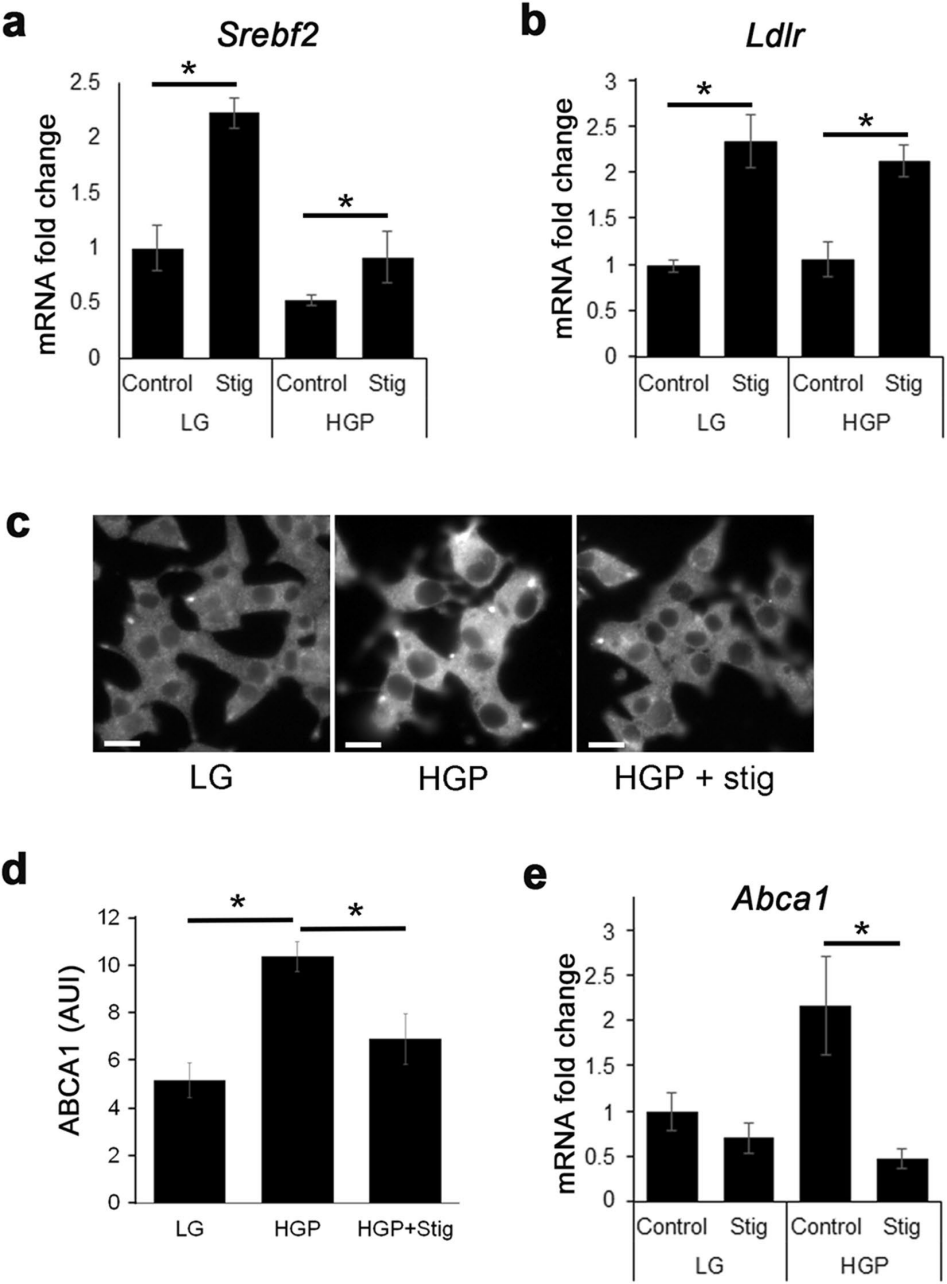
Stigmasterol promotes transcriptional changes associated with cholesterol homeostasis. INS-1 cells were incubated in LG or HGP, and treated with or without stigmasterol (Stig). (**a**,**b**) Transcription of *Srebf-2* (**a**) and *Ldlr* (**b**) measured by RT-PCR. (**c-e**) Immunohistochemistry (**c**) and quantification of ABCA1 expression (**d**) and *Abca1* transcription (**e**) levels under all conditions. Scale bars, 10 μm. *p < 0.05.

Because different phytosterols have cell-type specific effects on cholesterol regulatory genes^27^, we examined whether stigmasterol influences genes associated with cholesterol homeostasis in β-cells. Genes involved in cholesterol regulation are under the control of sterol regulatory element-binding protein 2 (SREBP2). The activation of SREBP2 is well characterized and is highly regulated by intracellular cholesterol levels. Low cholesterol levels are sensed in the ER by SCAP, which chaperones SREBP2 to the Golgi apparatus, where SREBP2 is cleaved by proteases, and is transported to the nucleus. The transcription of the SREBP2 gene *Srebf2* is under a feed forward mechanism, where the transcription of *Srebf2* is increased by SREBP2 activation^37^. Stigmasterol has been shown to interfere with SREBP processing^38^, which may elicit a compensatory response in *Srebf2* expression in stigmasterol-treated cells. As shown in Fig. 5a, the addition of stigmasterol to either LG or HGP treated INS-1 cells increased transcription of *Srebf2* (Stig vs Control). An SREBP2 target gene *Ldlr* ^39^, responsible for cholesterol uptake from LDL, was similarly upregulated (Fig. 5b). HGP + Stig treated cells had decreased cholesterol efflux transporter ABCA1 when compared with HGP treatment at both the protein and transcriptional level (Fig. 5c–e). The exact mechanism that causes cholesterol genes to change in cells loaded with phytosterols is unknown. It has been shown that it is the accumulation of stigmasterol, rather than changes in the cholesterol level, that is responsible for altered expression of cholesterol homeostasis genes^38^.

### Stigmasterol improves β-cell function and reduces β-cell apoptosis

**Figure 6:**
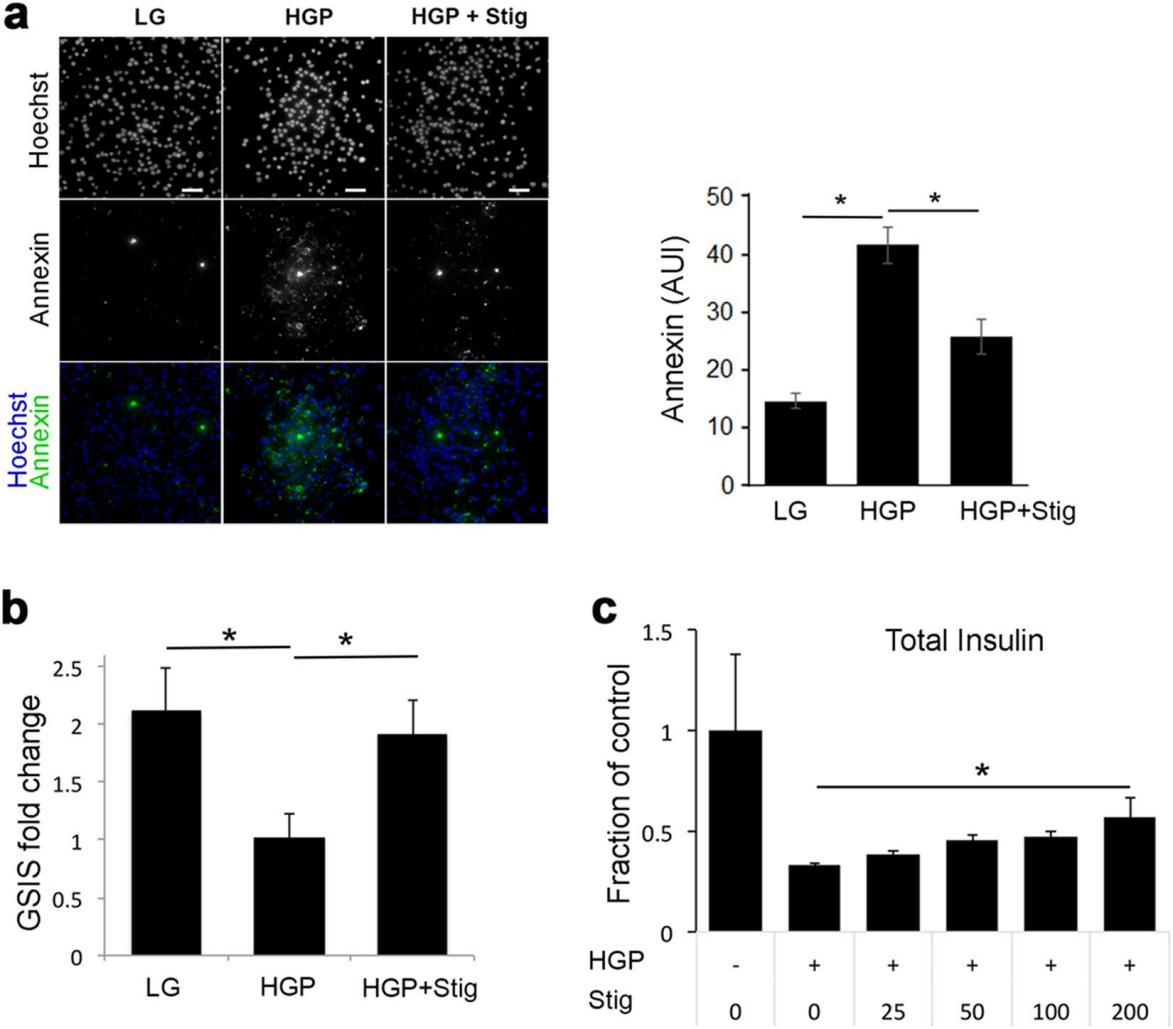
Stigmasterol prevents early apoptosis and impaired insulin secretion due to HGP. (**a**) Annexin V staining of INS-1 cells treated with LG, HGP and HGP + stig, along with Hoechst staining for cell counting. Scale bars, 50 μm. (**b**) GSIS of INS-1 cells treated with LG, HGP and HGP + stig. Results are presented as the ratio of insulin secretion at 20 mM glucose to insulin secretion at 2 mM glucose. Data are pooled from six different experiments. *p < 0.05. (**c**) Total insulin from INS-1 cells treated with HGP and varying concentrations of stigmasterol, normalized to cells with no HGP treatment. *p < 0.05, HGP vs. HGP + Stig treatment.

The direct effect of stigmasterol on β-cells is unclear. While Panda and colleagues reported a decrease in serum glucose accompanied by increased circulating insulin when stigmasterol was administered to mice^36^, others have shown no effect of stigmasterol feeding on glucose tolerance^40^. Glucolipotoxicity is known to induce β-cell death and reduce β-cell function^41^. We first examined the effect of stigmasterol on β-cell survival. External phosphatidylserine is a marker of early apoptosis, since phosphatidylserine is normally located on the inner leaflet of the plasma membrane, but is flipped to the outer membrane during apoptosis and necrosis^42^. Annexin V binds to phosphatidylserine; thus, increased annexin V staining is a measure of cell death. As shown in Fig. 6a, HGP treatment increased annexin V staining in INS-1 cells. The addition of stigmasterol partially blocked the increase in annexin V, indicating that stigmasterol prevents β-cell death due to glucolipotoxicity. Similar results were obtained with Western blotting of active caspase-3 (Supplementary Fig. 4), a marker for apoptosis^43^.

In order to examine the direct effects of stigmasterol on β-cell function, static GSIS assays were performed. Addition of stigmasterol rescued GSIS in cells treated with HGP (Fig. 6b). Under this condition, stigmasterol also exerted a beneficial effect of increasing total insulin in HGP treated INS-1 cells (Fig. 6c). Together these data indicate that stigmasterol treatment can preserve β-cell function and survival in the presence of glucolipotoxicity.

### Stigmasterol rescues glucose-stimulated actin reorganization

Actin reorganization plays an essential role in glucose-stimulated insulin secretion. Pancreatic β-cells display a dense actin web below the plasma membrane that may inhibit granule docking. Glucose-stimulated actin reorganization serves to remove the F-actin barrier and permit access of granules to the plasma membrane. When this process is inhibited, GSIS is reduced^44, 45^. In the next series of experiments, we tested the hypothesis that glucolipotoxicity inhibits GSIS through promoting actin polymerization induced by excess cholesterol and that this process could be prevented by stigmasterol.

**Figure 7:**
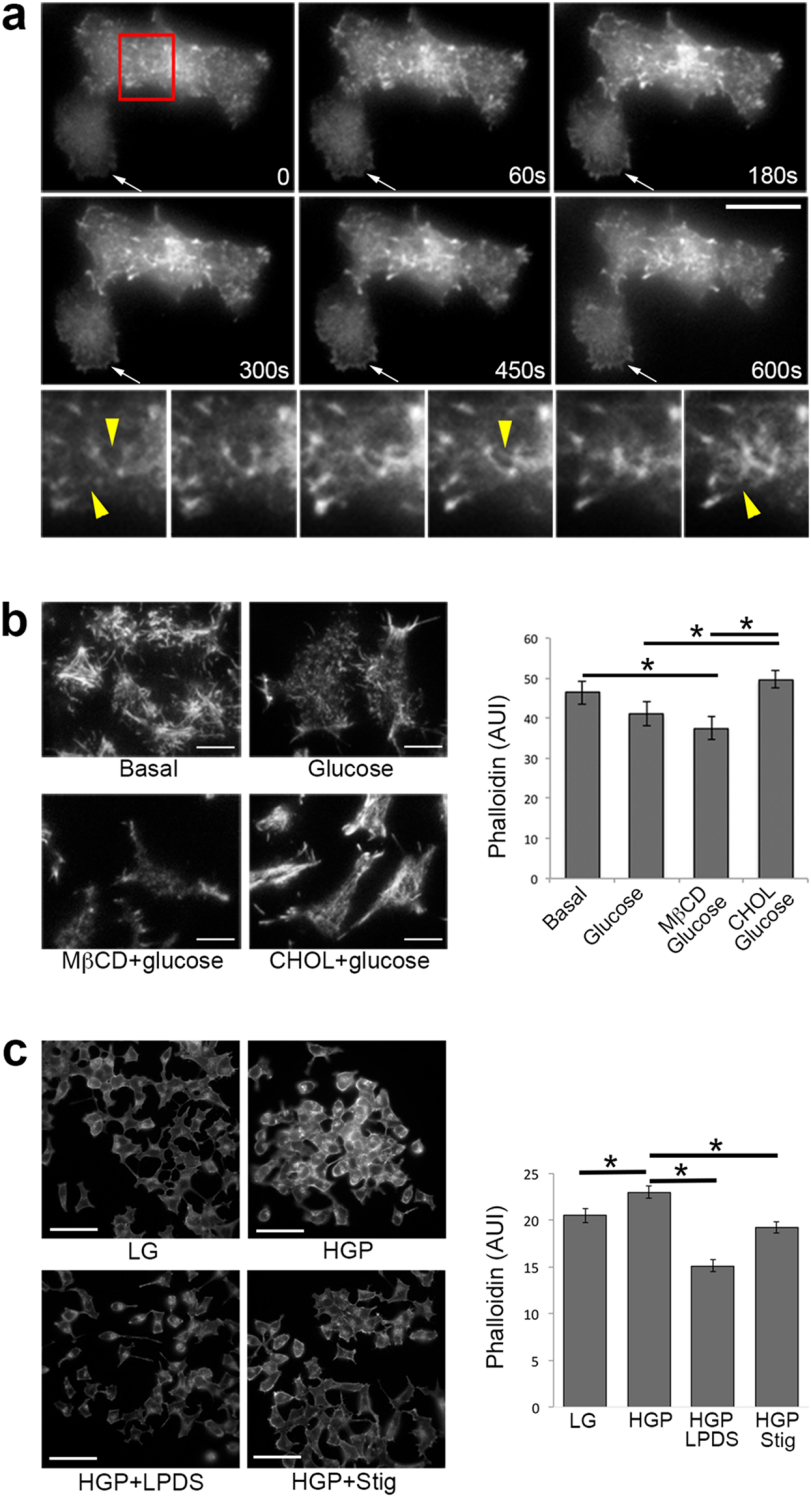
Stigmasterol reverses HGP-mediated actin polymerization. (**a**) Real-time polymerization of actin-GFP at the plasma membrane in MIN6 cells exposed to cholesterol loading. TIRFM images were taken every 2 s. The time points refer to the time after adding soluble cholesterol (CHOL). The lower panels show enlarged images of the red-boxed region indicated in the first frame. The arrow points to a cell in which rapid actin polymerization took place, especially around the cell periphery. Scale bars, 10 μm. (**b**) Fluorescence imaging and quantification of F-actin labeled with phalloidin in cholesterol treated cells. “Basal” cells were starved in 2 mM glucose for 2 h; “Glucose” cells were incubated with 20 mM glucose for 15 min, “MβCD” cells were incubated with 5 mM MβCD at 37 °C for 1 h, and “CHOL” cells were incubated with 5 mM CHOL for 1 h at 37 °C. 20 mM glucose was added to the last 15 min of cholesterol treatment. Scale bars, 10 μm. *p < 0.05. (**c**) Fluorescence imaging and quantification of F-actin labeled with phalloidin in HGP treated cells. INS-1 cells were treated for 72 h with LG, HGP, HGP + LPDS or HGP + Stig. Scale bars, 50 μm. *p < 0.05.

We previously showed that cholesterol overloading induces actin polymerization, leading to membrane protrusion, membrane ruffling, and cell expansion in cultured β-cells, suggesting a role of cholesterol in regulating cortical F-actin^46^. Here we extended our previous actin studies of fixed cells to live cell total internal reflection fluorescence microscopy (TIRFM) studies of real-time cortical actin dynamics in β-cells expressing actin-GFP. TIRFM was used to ensure that the focus was on cortical F-actin at the plasma membrane where insulin granule exocytosis took place. Within minutes after adding soluble cholesterol, formation of actin filaments was seen in both cells in Fig. 7a. Rapid actin polymerization occurred in the lower cell, evidenced especially by the thick actin ring around the rim (arrows). Enlarged images of the upper cell are presented in the bottom panels, which show that long actin filaments appeared from what were previously smaller fragments (arrowheads). This set of images provides evidence for a direct involvement of excess cholesterol in actin polymerization in cultured β-cells.

To further confirm that cholesterol regulates actin reorganization upon glucose stimulation, a process that is critical for GSIS, cells were first cholesterol depleted (by MβCD) or overloaded (by soluble cholesterol, “CHOL”), then stimulated with 20 mM glucose, fixed and stained using phalloidin, a probe specific for polymerized F-actin^47^ (Fig. 7b). Compared with basal unstimulated cells, glucose-stimulated cells displayed slightly less F-actin staining, consistent with the idea that glucose stimulates actin reorganization to remove the F-actin barrier and thus to facilitate insulin release^44, 45^. Cholesterol overloaded cells stimulated with glucose (“CHOL”), on the other hand, had a mesh-like layer of F-actin network and significantly increased amount of F-actin, compared with untreated cells stimulated with glucose. Quantification of F-actin content under the four conditions is presented in Fig. 7b. These data show that cholesterol is actively involved in the actin reorganization process during glucose stimulation.

Finally, we examined phalloidin staining in HGP-treated cells with or without stigmasterol. HGP treatment significantly increased F-actin in INS-1 cells, which was normalized by stigmasterol treatment (Fig. 7c). Cholesterol depletion by LPDS, serving as a positive control for cholesterol-mediated actin dynamics, also reduced polymerized actin. The results from Fig. 7 support a model that the crucial step of glucose-stimulated actin depolymerization in GSIS is inhibited by glucolipotoxicity-induced cholesterol accumulation and that stigmasterol rescues GSIS at least partially by increasing actin dynamics in β-cells.

## Discussion

The study presented here shows that stigmasterol protects pancreatic β-cells from glucolipotoxicity by preventing accumulation of free cholesterol and ROS, improving insulin secretion, increasing insulin content and decreasing markers for early apoptosis. We provide evidence that glucolipotoxicity stimulates ROS production, and that free radicals in turn promote cholesterol accumulation within β-cells. Stigmasterol treatment can normalize cellular cholesterol levels by reducing ROS production from glucolipotoxicity in β-cells. Indeed, stigmasterol can serve as a mild antioxidant or pro-oxidant to lipid peroxidation depending on the substrate^48^. Intraperitoneal injection of stigmasterol decreases lipid peroxidation and increases scavenger proteins in the livers of Ehrlich Ascites Carcinoma mice^49^. Stigmasterol did not increase *Abca1* expression in INS-1 cells, indicating that stigmasterol may work differently in β-cells than in macrophages^27^.

It has long been recognized that Type 2 diabetes is associated with disordered lipid homeostasis, including increased VLDL and decreased HDL^50^. Thus, perturbations in circulating cholesterol may be a predisposing factor for type 2 diabetes. In agreement with these clinical findings, excess cholesterol and disrupted cholesterol export can promote β-cell dysfunction^12, 51, 52^. HMG-CoA reductase inhibitors (statins) are widely used to decrease serum cholesterol to prevent cardiovascular diseases. Several meta-analyses indicate, however, that statins may actually increase diabetes risk^16–19^. *In vitro* studies also suggest that statin treatment can negatively affect β-cells. Statins increase basal insulin secretion in MIN-6 cells, lowering the fold change in insulin secretion upon glucose stimulation^53^. Atorvastatin decreased insulin content in rat islets and INS-1 cells^54^. It should be noted that these *in vitro* studies were performed in systems with normal cholesterol levels. Cholesterol extraction using MβCD from LDLR^−/−^ islets with elevated cholesterol reported an improvement in insulin secretion^55^. Therefore, depletion of β-cell cholesterol may be beneficial during times of cholesterol excess.

Studies on the link between statin usage and increased diabetes risk caution us against relying on statins to reduce cholesterol as a means to improve β-cell function under glucolipotoxicity. Few studies have focused on the effect of phytosterols on diabetes. Db/db mice represent a widely used animal model for obesity-induced diabetes. When they were fed Aloe Vera, they displayed decreased fasting blood glucose and HbA1c levels (a marker of blood glucose control) which were attributable to the phytosterol containing extracts^56^. Similarly, Zucker diabetic fatty rats exhibited increased glucose tolerance and decreased HbA1c levels when Aloe Vera derived phytosterols were administered orally^57^. Patients fed a phytosterol-enriched spread displayed a small, but significant, decrease in HbA1c levels^58^. One study looking at the effects of phytosterols on high fat fed mice reported that phytosterols did not improve glucose and insulin tolerance^40^, although β-cell function was not measured.

Cortical F-actin is dramatically increased in cholesterol-overloaded cells (Fig. 7), which could inhibit insulin granule exocytosis by several mechanisms. It may present a physical barrier, which impairs the ability of insulin granules to dock at the plasma membrane and reduces the size of the readily releasable pool of insulin granules. Indeed, an increase in the readily releasable pool of insulin granules has been suggested in cholesterol depleted cells^59^ and cholesterol overloading reduces the number of insulin granules docked at the plasma membrane^60^. Dense F-actin could restrict syntaxin 4 accessibility, which is required for insulin exocytosis^61^. Disruption of F-actin increases syntaxin 4 accessibility to VAMP2 by promoting dissociation of syntaxin 4 from F-actin, leading to increased GSIS^62^. Because cholesterol overloading results in cortical F-actin polymerization, it is likely that cholesterol alteration would interfere with the interaction between syntaxin 4 and F-actin, thereby affecting the required function of syntaxin 4 in GSIS. One potential mechanism for the involvement of stigmasterol in rescuing GSIS from HGP-treated β-cells is reducing the detrimental effect of excess cholesterol on glucose-stimulated actin reorganization.

In conclusion, our study shows beneficial effects of stigmasterol treatment on diabetic β-cells. Further studies will be needed to determine if oral intake of stigmasterol prevents β-cell dysfunction in diabetes.

## Methods

### Cell culture

INS-1 832/13 (INS-1) cells^63^ were cultured in RPMI-1640 supplemented with 10% fetal bovine serum (FBS), 100 U/ml penicillin, 100 μg/ml streptomycin, 10 mM HEPES, 2 mM L-glutamine, 1 mM sodium pyruvate, and 50 μM mercaptoethanol, at 37 °C in 5% CO_2_. MIN6 cells were grown in DMEM supplemented with 100 U/ml Penicillin, 100 µg/ml Streptomycin, 10% FBS, 2 mM L-glutamine and 50 µM 2-mercaptoethanol, at 37 °C in 5% CO_2_. Unless otherwise indicated, all experiments were performed using INS-1 cells. For cell culture treatments, INS-1 cells or human islets were incubated in normal growth media with 0.5% BSA (LG), growth media supplemented to a final concentration of 30 mM glucose, 0.5 mM palmitate-BSA complex (HGP), or HGP supplemented with stigmasterol (HGP + stig) for 72 h. For stigmasterol supplementation, stigmasterol stocks were made from stigmasterol powder (95%, Sigma) dissolved in 100% ethanol at a final concentration of 5 mg/ml. Unless otherwise stated, 50 μg/ml stigmasterol was used. Where indicated, 2,2′-Azobis(2-amidinopropane) dihydrochloride (AAPH) was dissolved in FBS-free RPMI media to indicated concentrations. Human islets were commercially obtained from InSphero (Zurich, Switzerland) and cultured according to manufacturer instructions.

### Materials

Alexa546-phalloidin, lipidTOX red, actin-GFP and carboxy-H_2_DFFDA were from Thermo Fisher Scientific. AAPH was from Cayman Chemical. Rabbit anti-ABCA1 was from Novus Biologicals. Antibodies to active Caspase-3 and GAPDH were from Abcam. Filipin, mevinolin, N-acetyl-l-cysteine (NAC), MβCD, water-soluble cholesterol (CHOL) and all other chemicals were from Sigma. KRBH buffer (128.8 mM NaCl, 4.8 mM KCl, 1.2 mM KH_2_PO_4_, 1.2 mM MgSO_4_, 2.5 mM CaCl_2_, 5 mM NaHCO_3_, 10 mM HEPE, pH 7.4) was used for all experiments.

### Palmitate/BSA complex preparation

Palmitate solution was prepared as described previously^64^ by making a 20 mM palmitate stock in 0.1 M NaOH and heating to 70 °C for 30 min. Palmitate stock was added to 10% BSA in RPMI-1640 for a final concentration of 4 mM palmitate complex and heated at 55 °C for 30 min with occasional shaking.

### Cholesterol manipulation

Cholesterol depletion using methyl-β-cyclodextrin (MβCD) was done by incubating cells with 5 mM MβCD at 37 °C for 1 h; to cholesterol overload, cells were incubated with 5 mM soluble cholesterol (CHOL, 1 g contains approximately 40 mg cholesterol) at 37 °C for 1 h. LPDS treatment was done by culturing cells in normal growth medium with 10% FBS replaced by lipoprotein deficient serum (LPDS medium). Mevinolin treatment was done by including 1 μM mevinolin in LPDS medium. HDL treatment was done by including 50 μg/ml HDL in LPDS growth medium.

### Glucose stimulated insulin secretion

Static glucose stimulated insulin secretion was measured as previously described^65^. Briefly, INS-1 cells were incubated in glucose-free RPMI-1640 culture medium for 2 h. Cells were starved for 1 h in glucose-free KRBH buffer. INS-1 cells were incubated in 2 mM glucose in KRBH for 1 h (basal samples), followed by 1 h in 20 mM glucose in KRBH (glucose-stimulated samples). Incubations were performed at 37 °C in an air incubator. Total insulin samples were taken after 1% Triton X-100 extraction. To measure the basal and stimulated insulin secretion separately, secreted insulin was normalized to total protein. Insulin samples were measured by insulin ELISA kits (Alpco Diagnostics).

### Fluorescence Microscopy

Cells were grown in imaging dishes similar to MatTek dishes. Wide-field fluorescence microscopy utilized a Leica DMIRB microscope (Leica Mikroscopie und Systeme GmbH, 35578 Wetzlar, Germany) equipped with a Princeton Instruments (Princeton, NJ, USA) cooled charge coupled device (CCD) using MetaMorph Imaging System software (Molecular Devices). Images were acquired using an oil-immersion objective (×40). For filipin and lipidtox staining, INS-1 cells were fixed in 1% paraformaldehyde for 20 minutes then incubated in filipin (50 μg/ml) or lipidtox (1:1000 dilution) for 45 min. Filipin and lipidtox images were captured using UV and rhodamine filters, respectively. Details of filipin staining and imaging are described previously^66^. For carboxy-H_2_DFFDA fluorescence, INS-1 cells were incubated in 20 μM carboxy-H_2_DFFDA for 1 h. Cells were washed and counterstained with Hoechst 33342 to label the nuclei for cell counting. For phalloidin staining, cells were fixed with 4% paraformaldehyde for 20 min, permeabilized with 0.1% Triton X-100 for 5 min, and stained with Alexa 546-phalloidin. All staining was done at room temperature.

### RT-PCR

Total RNA from INS-1 cells was extracted from tissues using the Purelink® RNA kit (Thermo Fisher Scientific). Total RNA (1 µg) was reverse transcribed using the SuperScript® Reverse Transcription system (Thermo Fisher Scientific). Real time PCR was performed on the cDNA on a Bio-Rad iCycler using SYBR green mastermix (Thermo Fisher Scientific). Thermal cycling conditions were: 95 °C for 5 min, (30 seconds 95 °C, 30 seconds 60 °C, 1 minute 52 °C) for 40 cycles, followed by a melt curve. Gene expression was expressed as the ratio between the expression of the target gene and GAPDH using the ΔΔCt method. Primers (Integrated DNA Technologies) used: Gapdh (forward 5′-actcccactcttccaccttc-3′, reverse 5′-tcttgctcagtgtccttgc-3′), Abca1 (forward 5′-aacagtttgtggcccttttg-3′, reverse 5′-agttccaggctggggtactt-3′), Ldlr (forward 5′-cagcctagaggggtaaactg-3′, reverse 5′-tagcataccatcagggcaag-3′), Srebf-2 (forward 5′-cgaactgggcgatggatgag-3′, reverse 5′-gacaaactgtagcatctcgtcg-3′).

### TIRFM

Transient transfections were performed with Lipofectamine 2000 (Thermo Fisher Scientific) according to the manufacturer’s protocol and cells were cultured for 48 h prior to microscopy. Actin-GFP-expressing MIN6 cells grown in MatTek dishes were kept in an Air-therm (WPI) temperature-regulated environmental box at 37 °C throughout the imaging experiments. Cells were imaged for 2 min to establish basal baseline. A 10 × concentrated stock of soluble cholesterol (CHOL) was then added to the edge of the MatTek dish on the microscope stage. TIRFM was performed using an Olympus objective-type IX-70 inverted microscope fitted with a 60×/1.45 NA TIRFM lens (Olympus), controlled by Andor iQ software (Andor Technologies), and detected with a back-illuminated Andor iXon 897 EMCCD camera (512 × 512, 14 bit; Andor Technologies). The depth of the evanescent field was calculated to be 98 nm^67^.

### Western blotting

Total cell lysates were prepared, and protein concentration was determined by BCA (Thermo Scientific). Samples were immunoblotted against active caspase 3 (1:1000) and GAPDH (1:2000) at 4 °C overnight. Secondary antibodies conjugated with HRP were incubated for 1 h at room temperature. The membranes were then developed by ECL (Thermo Scientific), according to the manufacturer’s instructions.

### Image quantification and statistics

All images were analyzed using MetaMorph software (Molecular Devices). Images were background subtracted and either quantified as individual cells or whole fields normalized to cell counts. Unless otherwise indicated, each data point represents the average of three independent experiments. Data are presented as the mean ± SEM. Statistical significance was analyzed using unpaired Student’s *t* tests for comparison between two groups and one-way repeated measures ANOVA with Post-hoc comparisons using the Tukey HSD test for data from more than two groups, with *p*-values less than 0.05 deemed significant.

### Data availability

All data generated or analyzed during this study are included in this published article (and its Supplementary Information files).

## Electronic supplementary material

Supplementary figures
