# Supplementary material for: Stigmasterol prevents glucolipotoxicity induced defects in glucose-stimulated insulin secretion
Source: Sci Rep. 2017 Aug 25;7:9536. doi: 10.1038/s41598-017-10209-0 (PMC5573401; doi:10.1038/s41598-017-10209-0)
Supplement: Supplementary file 1 — Supplementary figures [file 41598_2017_10209_MOESM1_ESM.pdf]

Stigmasterol prevents glucolipotoxicity induced defects in glucose-stimulated insulin secretion

Meliza G. Ward, Ge Li, Valéria C. Barbosa-Lorenzi, Mingming Hao*

Department of Biochemistry, Weill Cornell Medical College, New York, NY 10065

*Corresponding author:

Department of Biochemistry, E-210, Weill Cornell Medical College, 1300 York Ave., New York, NY 10065, USA. Phone: 212.746.6562. Fax: 212.746.8875. E-mail: mhao@med.cornell.edu

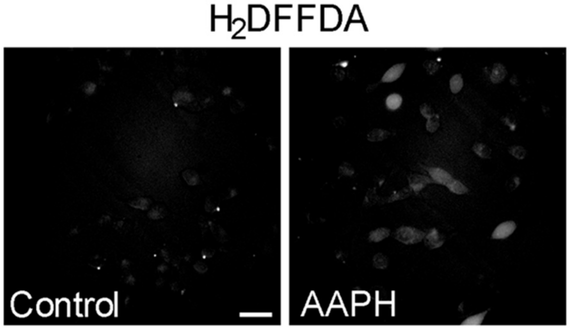

Supplementary Figure 1. AAPH induces ROS production in INS-1 cells. INS-1 cells were incubated for 1 h with 5 mg/ml AAPH and imaged using carboxy-H_2DFFDA. Scale bar, 15 μm.

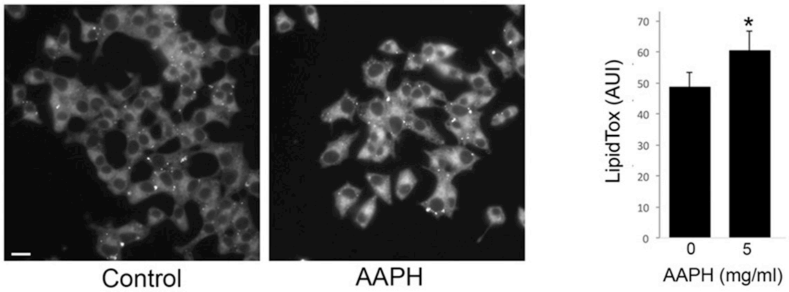

Supplementary Figure 2. ROS induces neutral lipid accumulation in INS-1 cells. Lipidtox staining of INS-1 cells incubated for 1 h with 5 mg/ml AAPH in serum free media. Scale bar, 15 μm. Lipidtox intensity per cell was quantified. *p<0.05.

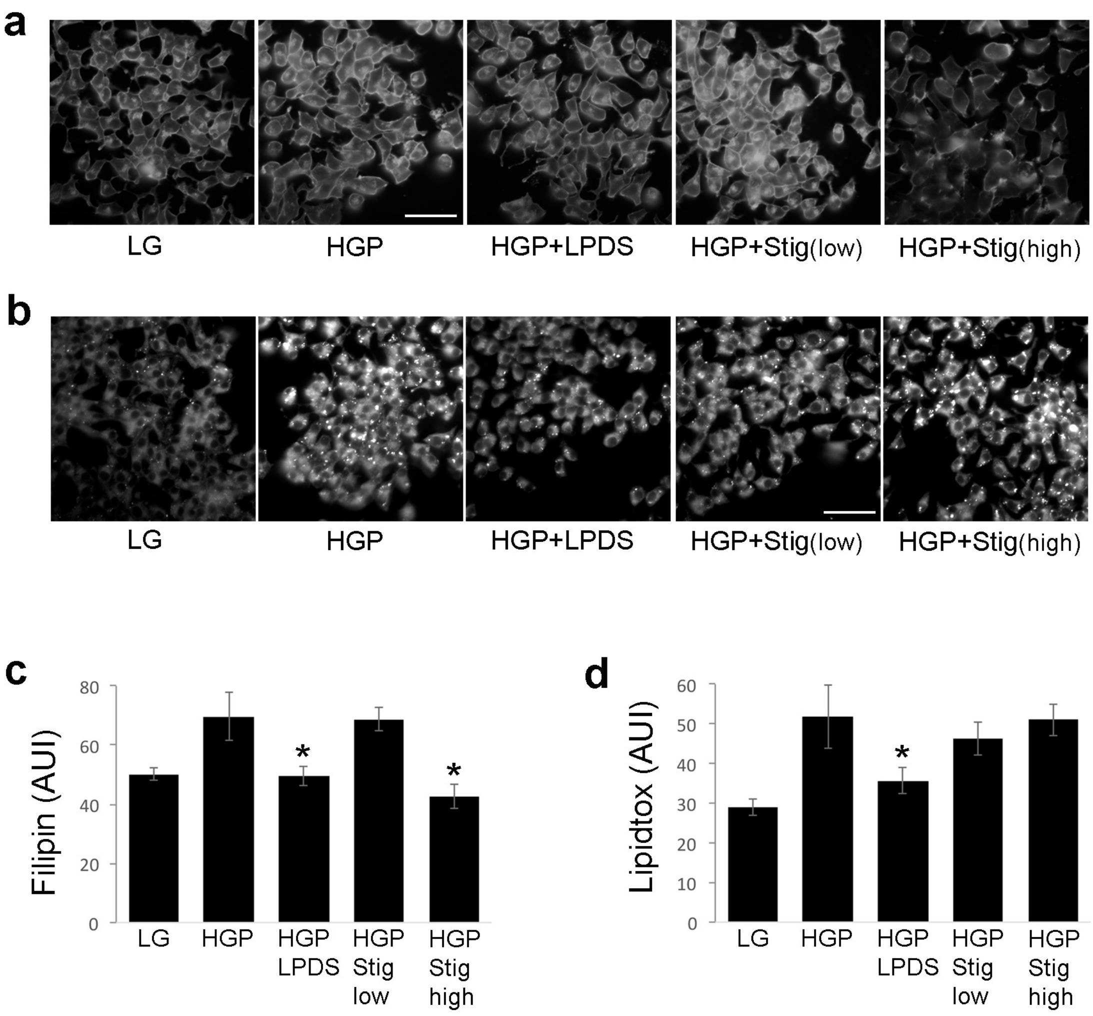

Supplementary Figure 3. Reversal of HGP-induced cholesterol increase by LPDS and stigmasterol. INS-1 cells treated for 72 h with LG, HGP, HGP+LPDS or HGP+stigmasterol (Stig) were stained with filipin (a) or lipidtox (b). Low and high stigmasterol concentration refers to 12.5 μg/ml and 50 μg/ml, respectively. Filipin (c) and lipidtox (d) fluorescence was quantified. Scale bars, 50 μm. *p<0.05 against HGP.

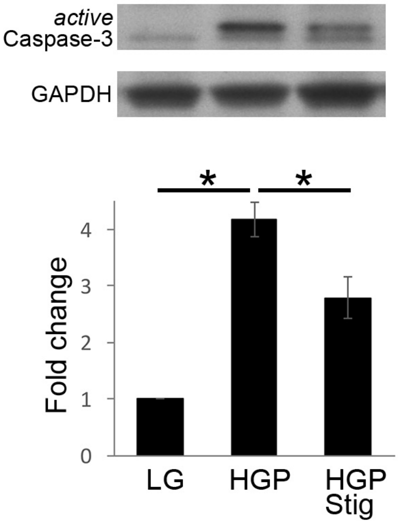

Supplementary Figure 4. Stigmasterol reduces HGP-mediated apoptosis indicated by decreased active caspase-3. Cells were treated as indicated, lysed and immunoblotted for active (cleaved) caspase-3. Equal amounts of total protein were loaded and GAPDH was used as loading control. *$p<0.05$ against HGP.
